# Three-dimensional-printed custom-made hemipelvic endoprosthesis for the revision of the aseptic loosening and fracture of modular hemipelvic endoprosthesis: a pilot study

**DOI:** 10.1186/s12893-021-01257-5

**Published:** 2021-05-26

**Authors:** Jie Wang, Li Min, Minxun Lu, Yuqi Zhang, Jingqi Lin, Yi Luo, Yong Zhou, Chongqi Tu

**Affiliations:** 1 Department of Orthopaedics, Orthopaedic Research Institute, West China Hospital, Sichuan University, No. 37 Guo Xue Xiang, Chengdu, 610041 People’s Republic of China; 2 Bone and Joint 3D-Printing and Biomechanical Laboratory, Department of Orthopaedics, West China Hospital, Sichuan University, Chengdu, People’s Republic of China

**Keywords:** 3D-printed, Custom-made, Modular, Hemipelvic endoprosthesis, Revision

## Abstract

**Background:**

The aims of this pilot study were (1) to assess the efficacy of 3D-printed custom-made hemipelvic endoprosthesis in restoring the natural location of acetabulum for normal bodyweight transmission; (2) to evaluate the short-term function of the revision with this endoprosthesis and (3) to identify short-term complications associated with the use of this endoprosthesis.

**Methods:**

Between February 2017 and December 2017, seven patients received revision with 3D-printed custom-made hemipelvic endoprosthesis. The body weight moment arm (BWMA) and cup height discrepancy (CHD) after primary and revisional surgery were analyzed to assess acetabulum location with plain radiography. After a median follow-up duration of 29 months (range 24–34), the function was evaluated with the Musculoskeletal Tumor Society (MSTS-93) score and Harris hip score (HHS). Complications were recorded by chart review.

**Results:**

The acetabulum locations were deemed reasonable, as evaluated by median BWMA (primary vs. revision, 10 cm vs. 10 cm) and median CHD (primary vs. revision, 10 mm vs. 8 mm). The median MSTS-93 score and HHS score were 21 (range 18–23) and 78 (range 75–82) after the revision. No short or mid-term complication was observed in the follow-up of this series.

**Conclusions:**

Revision with 3D-printed custom-made hemipelvic endoprostheses benefited in reconstructing stable pelvic ring and natural bodyweight transmission for patients encountering the aseptic loosening and fracture of modular hemipelvic endoprosthesis. The revision surgery and appropriate rehabilitation program improved patients’ function to a median MSTS score of 22 and pain-free ambulation. The incidence of the complications was low via this individualized workflow.

## Background

Hemipelvic reconstruction after type I + II resection is still challenging, and various methods have been proposed, such as no reconstruction, hip transposition, allograft, modular endoprosthesis, and custom-made endoprosthesis [1–7]. Between two endoprostheses, the modular endoprosthesis is regarded as the prior solution to provide good initial stability and relatively rapid restoration of function by some surgeons since the early 1970s [2, 8–11]. Recently, with advances in imaging and systemic therapies, patients survive longer from malignancies after hemipelvic limb-salvage surgery [12–14]. As a result, mechanical complications, caused by poor osseointegration, persistent micromotion, and defective bodyweight transmission, have become a major problem and require a proper solution [10–12].

The treatments for aseptic loosening and fracture are seldom described. So far, reported therapeutic options include a conservative method, partial retrieval of the endoprosthesis, total retrieval of the endoprosthesis followed by flail hip, and total retrieval of endoprosthesis followed by revision [2, 10, 11, 15]. The conservative method is a mainstream treatment but failed endoprosthesis can gradually destruct host bone during daily activities [15]. Therefore, surgical intervention is ultimately inevitable for some patients. Partial retrieval of the endoprosthesis is suitable for endoprosthesis fracture without instability, such as retrieving fractured parts for pubic plate fracture [2, 10, 11]. Loosening with instability should consider the total retrieval of the endoprosthesis, and the residual bone defect requires appropriate reconstruction [10, 11, 16]. Indeed, the flail hip can restore partial lower-limb function, but limb length discrepancy and extended immobilization duration are unacceptable for some patients. Theoretically, revision with a proper endoprosthesis is another potential method by offering immediate support to restore lower-limb function.

As two standard options in primary hemipelvic reconstruction, the modular hemipelvic endoprosthesis and the 3D-printed custom-made hemipelvic endoprosthesis vary in the compatibility to host bone. The modular endoprosthesis is pre-manufactured with a component design, allowing intraoperative adjustment according to diversified bone defects; whereas the matching degree is usually sacrificed when connecting modular hemipelvic endoprosthesis with uniform appearance to host pelvic bone with an irregular shape [2, 11, 12]. In contrast, a 3D-printed custom-made hemipelvic endoprosthesis is individually designed based on a patient’s data [17]. The compatibility can be ensured with advanced design software and precise manufacturing techniques. Additionally, a 3D-printed custom-made hemipelvic endoprosthesis can be fabricated as one component to reduce the junction part's possibly mechanical failure. Therefore, the 3D-printed custom-made hemipelvic endoprosthesis is considered more proper for complex hemipelvic revision. As far as we know, the application of 3D-printed custom-made hemipelvic endoprosthesis in revision is not sufficiently represented in the literature. Such workflow is highly demanding due to the destruction and migration of host bone, deformation of the medullary cavity, scar tissue generation, and potential failure during device retrieval.

We recently designed a series of 3D-printed custom-made hemipelvic endoprosthesis for patients with aseptic loosening and fracture of modular hemipelvic endoprosthesis, and a satisfactory clinical outcome was observed. The aims of this pilot study were (1) to assess the efficacy of 3D-printed custom-made hemipelvic endoprosthesis in restoring natural location of acetabulum for normal bodyweight transmission; (2) to evaluate the short-term function of the revision with this endoprosthesis and (3) to identify short-term complications associated with the use of this endoprosthesis.

## Methods

### Patients

**Fig. 1:**
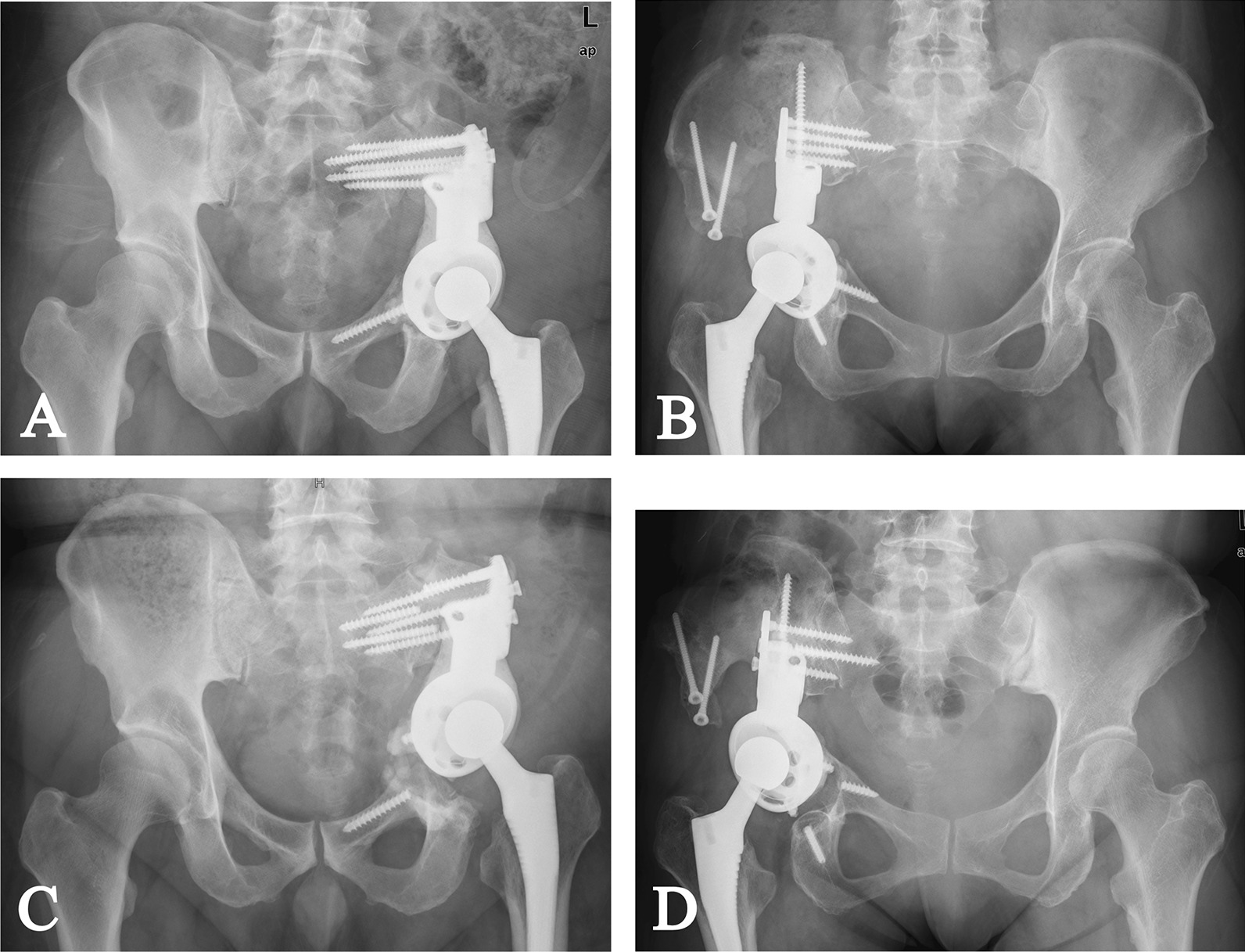
**A**, **B** Two AP plain radiographs of (**A**) a 60-year-old male with solitary plasmacytoma (Patient 1) and (**B**) a 43-year-old female with chondrosarcoma (Patient 2) after the primary surgery, which show reconstruction with modular hemipelvic endoprosthesis; **C**, **D** Two AP plain radiographs of (**C**) patient 1 and (**D**) patient 2 before revision, which show aseptic loosening, screw fracture and migration of modular hemipelvic endoprosthesis

Between February 2017 and December 2017, seven patients with aseptic loosening and fracture of modular hemipelvic endoprosthesis (Chunli Co., Ltd., Tongzhou, Beijing, China) received total retrieval of modular hemipelvic endoprosthesis and revision with 3D-printed custom-made hemipelvic endoprosthesis. The indications for revision with 3D-printed custom-made endoprosthesis were (1) aseptic loosening with instability; (2) aseptic loosening with severe pain; (3) migrated endoprosthesis endangering essential structures around the endoprosthesis; (4) willing to take the potential risks of the 3D-printed custom-made endoprosthesis. There were four males and three females with a median age of 49 years (range 25–60). Totally, five patients had type I + II resection, and two patients had type I + II + III resection, and all patients received en bloc resection and reconstruction with modular hemipelvic endoprosthesis and construct femoral head autografting as their primary treatment [1]. After primary surgery, all patients underwent plain radiograph (PR) of the pelvis before discharge and monthly in the first three months and trimonthly after that (Fig. 1A, B). 3D-computerized tomography (3D-CT) of the pelvis was performed before discharge and yearly after the operation (Table 1).

**Table 1:** The demographics of the seven patients received revision with 3D-printed custom-made hemipelvic endoprosthesis

Patient	Age (year)	Gender	Resection classification*	Diagnosis	Follow-up (P, month)	Follow-up (R, month)	Oncologic outcome
1	60	M	Type I + II	Plasmacytoma	17	26	NED
2	42	F	Type I + II	Chondrosarcoma	54	29	NED
3	51	M	Type I + II + III	Plasmacytoma	36	32	NED
4	30	M	Type I + II	ASTS	16	27	AWD
5	54	F	Type I + II	MSC	51	34	NED
6	49	M	Type I + II + III	Plasmacytoma	73	24	NED
7	25	F	Type I + II	Osteosarcoma	27	31	NED
Median	49				36	29	

*ASTS* Alveolar soft tissue sarcoma, *MSC* Metastatic squamous carcinoma, *P* Primary surgery, *R* Revision, *NED* No evidence of disease, *AWD* Alive with disease

*According to Enneking and Dunham

Initial diagnoses were solitary plasmacytoma in three patients, chondrosarcoma in one, solitary metastatic squamous carcinoma in one, alveolar soft tissue sarcoma in one, and osteosarcoma in one. Patients with solitary plasmacytoma received chemotherapy (melphalan, prednisone) for five circles after primary surgery. Pulmonary metastasis occurred in patient 4 with alveolar soft tissue sarcoma before the primary surgery and received targeted therapy regularly after primary surgery with Apatinib (500 mg, q.d.). The patient with osteosarcoma received chemotherapy (doxorubicin, cisplatin) for nine circles after primary surgery. The median follow-up duration between primary and revision surgery was 36 months (range 16–73), and the median follow-up duration from revision to latest follow-up was 29 months (range 24–34). Six patients were no evidence of disease, and one patient was alive with disease. No patient was lost to follow-up (Table 1).

This study was approved by the Ethical Committee of our institution. Written informed consent to participate in this study was obtained from all the patients.

### Endoprosthesis design and fabrication

**Fig. 2:**
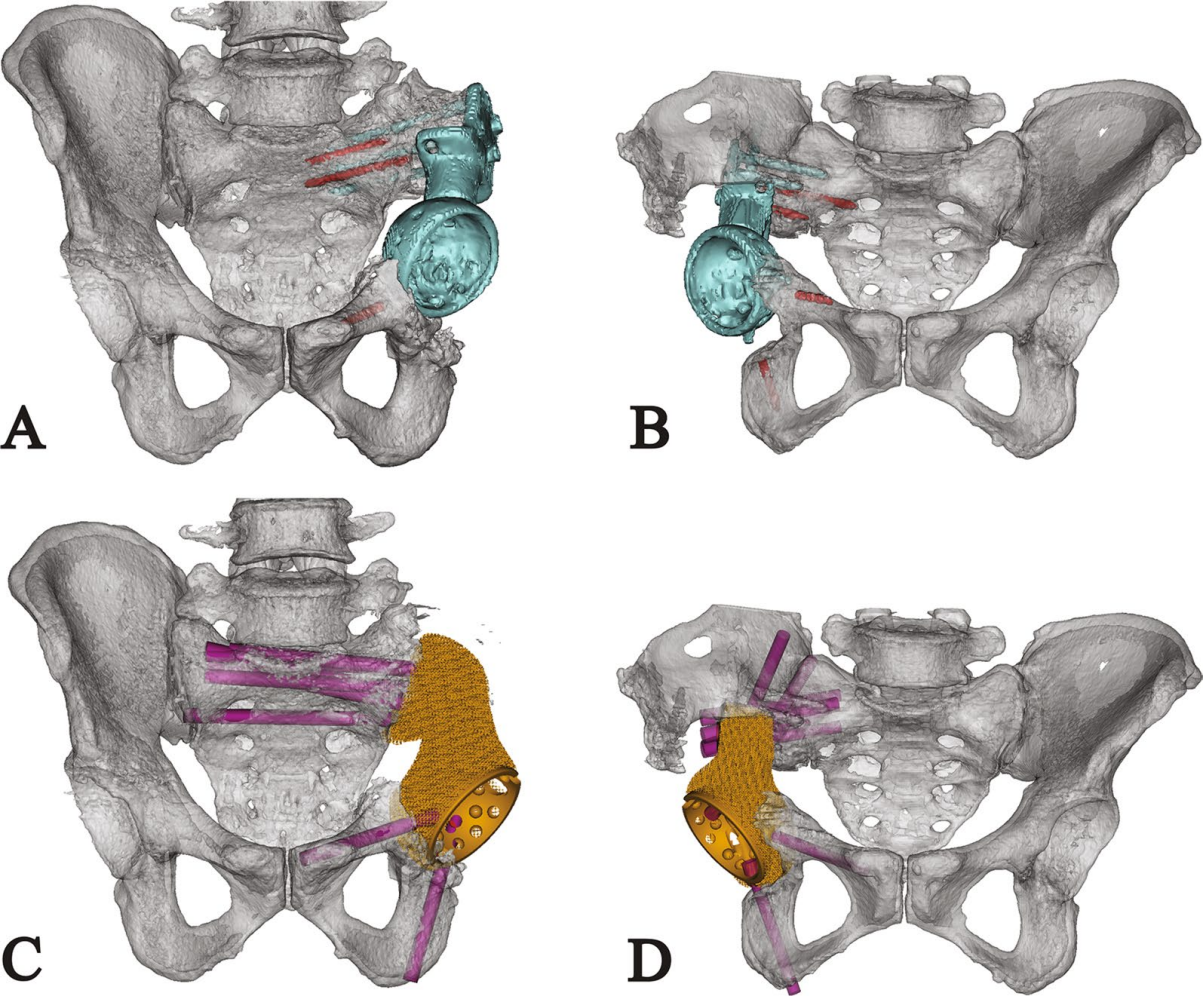
**A**, **B** Preoperative simulation shows endoprosthesis migration (blue) and broken screws (red) in (**A**) patient 1 and (**B**) patient 2; **C**, **D** Endoprosthesis design and screw fixation of (**C**) patient 1 and (**D**) patient 2 are shown

Before endoprosthesis design, PR and 3D-CT with metal artifact reduction technique of the pelvis were performed (Fig. 1C, D). The CT data were used to build virtual 3D models in the Mimics V20.0 software (Materialise Corp., Leuven, Belgium) after assessing and reducing the remaining artifact. Then the models of the pelvis were smoothened to show residual sclerotic bone clearly, and models of the fractured screw were highlighted (Fig. 2A, B).

All the endoprostheses for revision were designed by our clinical team and fabricated by Chunli Co., Ltd., Tongzhou, Beijing, China. With a streamlined outline, the endoprosthesis matched the bone defect, restored a continuous natural arcuate line, and rebuilt the intact pelvic ring (Fig. 2C, D). The endoprosthesis was composed of a porous structure with 600 µm-pore-size and 70%-porosity and a continuous “arc-like” solid supporting structure extending along the arcuate line and connecting other solid structures such as screw holes, the acetabulum, and pubic ramus [18]. If the cup height discrepancy (CHD) is under 20 mm, the artificial cup would be placed at the same height as the unaffected acetabulum. Otherwise, the artificial cup would descend 20 mm from the migrated cup. The acetabular anteversion and inclination were designed as 25° and 45°, respectively. Thereafter, the pubic screws were usually inserted from the acetabulum and through the medullary cavity of the pubis; the alignment of sacral screws was modified as obliquely upward, matching the arcuate line to the sacral promontory. Meanwhile, all the sacral screws were designed to avoid the tunnels of primarily inserted screws.

**Fig. 3:**
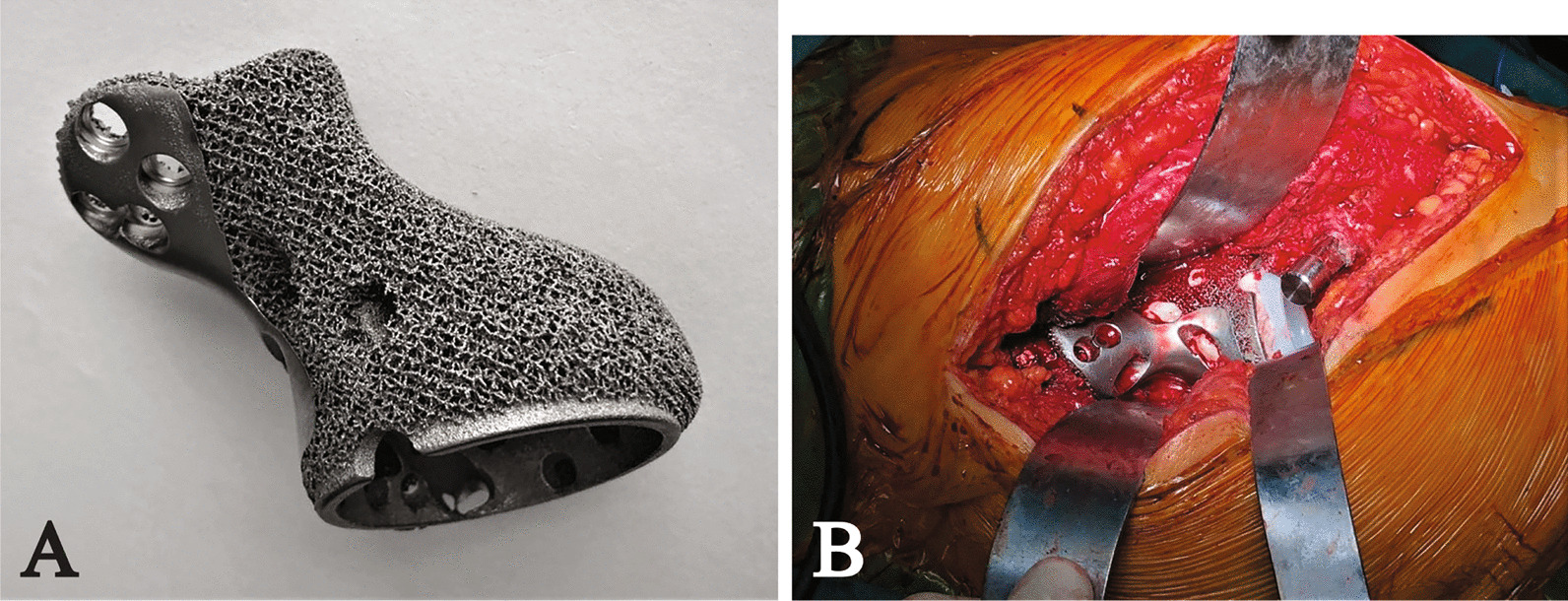
**A** The image shows lateral view of the endoprosthesis of patient 2; **B** The image shows the posterior approach and precise endoprosthesis implantation of patient 2

The endoprosthesis was fabricated using electron beam melting technique (ARCAM Q10plus, Mölndal, Sweden) (Fig. 3A). While the plastic endoprosthesis models were fabricated using stereolithography appearance technique (UnionTech Lite 450HD, Shanghai, China). The design and manufacturing of endoprosthesis and plastic models cost approximately US $6500.

### Surgical techniques

**Fig. 4:**
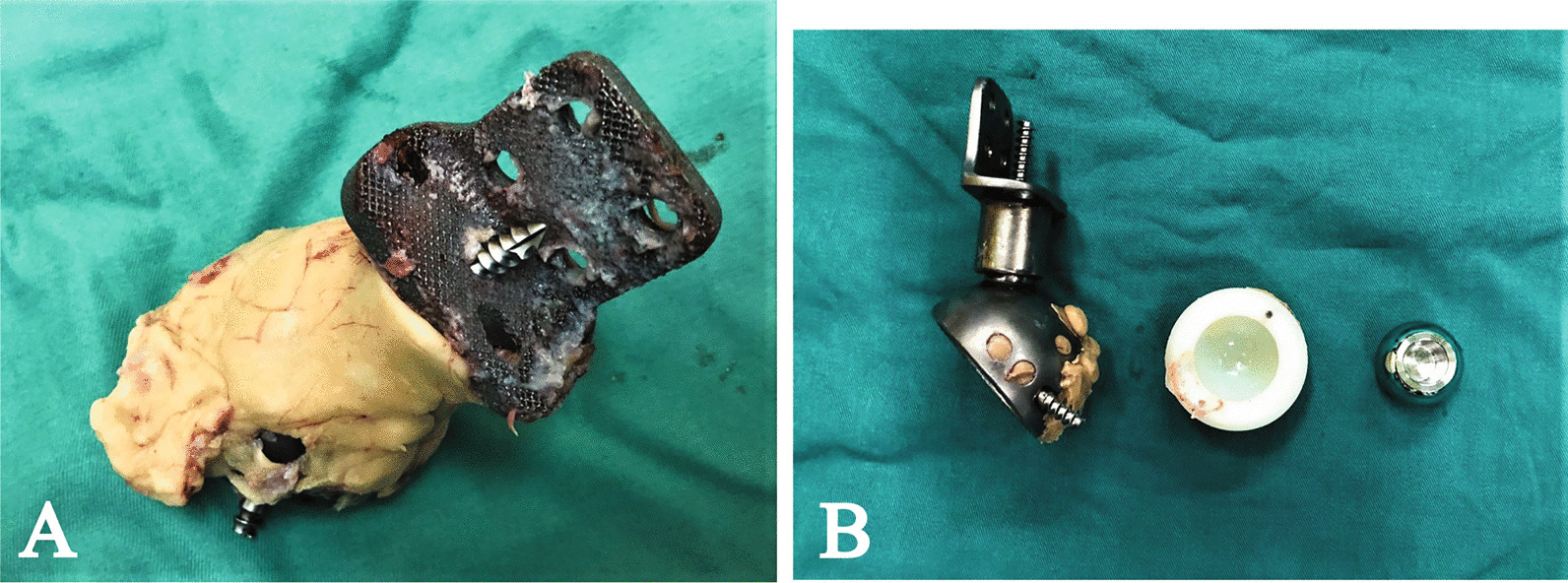
**A**, **B** The explanted modular hemipelvic endoprostheses show (**A**) fibrous tissue ingrowth however no osseointegration in patient 1 and (**B**) no osseointegration and the wear of acetabular liner striking in patient 2

All surgeries were performed by the same senior surgeon. For the patients with good preservation of the iliac crest, the posterior approach was applied, while for the patients with no iliac crest, combined posterior iliac and Smith-Petersen approach was repeated. Besides, an additional inguinal approach was prepared for retrieving the pubic component. Among seven patients, reduced primary approaches were used in four patients, while a new posterior approach was applied in three patients (Fig. 3B). After exposing the primary endoprosthesis, the dislocation of the hip joint was performed. And then, polyethylene liner and bone cement were removed to expose screw tails inside the acetabulum component (Fig. 4). The screws were retrieved by various instruments and operations to loosen the endoprosthesis. For the screw can’t be retrieved, the tail outside the host bone was cut to ensure no disruption to the following procedures. Then the bursa covering primary modular endoprosthesis was all excised by electrotome. Thereafter, the plastic implant trial and reduction simulation were performed to confirm the precise fit. The criteria of qualifying compact fit included the good connection of bone-implant interfaces, stable endoprosthesis while pushing the acetabulum, and well reconstruction of acetabular anteversion and inclination. The following steps were squirtile lavage, wound soak with 10% povidone-iodine solution for 3 min, and another squirtile lavage.

During reconstruction, the acetabular reamer was used to remove the fibrous tissue and sclerotic bone at interfaces to expose underneath trabecula, and the crushed bone was collected for bone autografting. Fixing to residual pubis or acetabulum was the prior step. The residual pubic ramus was checked before drilling tunnels to ensure the screw alignment. The same procedure was undertaken if ischium was preserved. Thereafter, the proximal interface connecting to the sacrum might be separated from the sacrum due to host bone migration. The reduction of endoprosthesis was performed to match two edges of host bone and endoprosthesis. After that, endoprosthesis was fixed to the sacrum by cancellous screws as preoperative simulation. A constrained acetabular component was then cemented into the endoprosthetic acetabulum, and then hip component reduction was performed. Before bone grafting with autograft or artificial bone, the wound was flushed by pulsing squirt gun, soaked by the 10% povidone-iodine solution. After that, the separated muscles were reconstructed, and the wound was sutured tightly layer by layer.

The median intraoperative time and blood loss were 210 min (range 180–240) and 600 ml (range 300–900), respectively.

### Postoperative management

The postoperative management was similar to those described in our previously published study [18]. The patient undertook two tests to evaluate hip muscle strength and determine their personalized rehabilitation program.

**Fig. 5:**
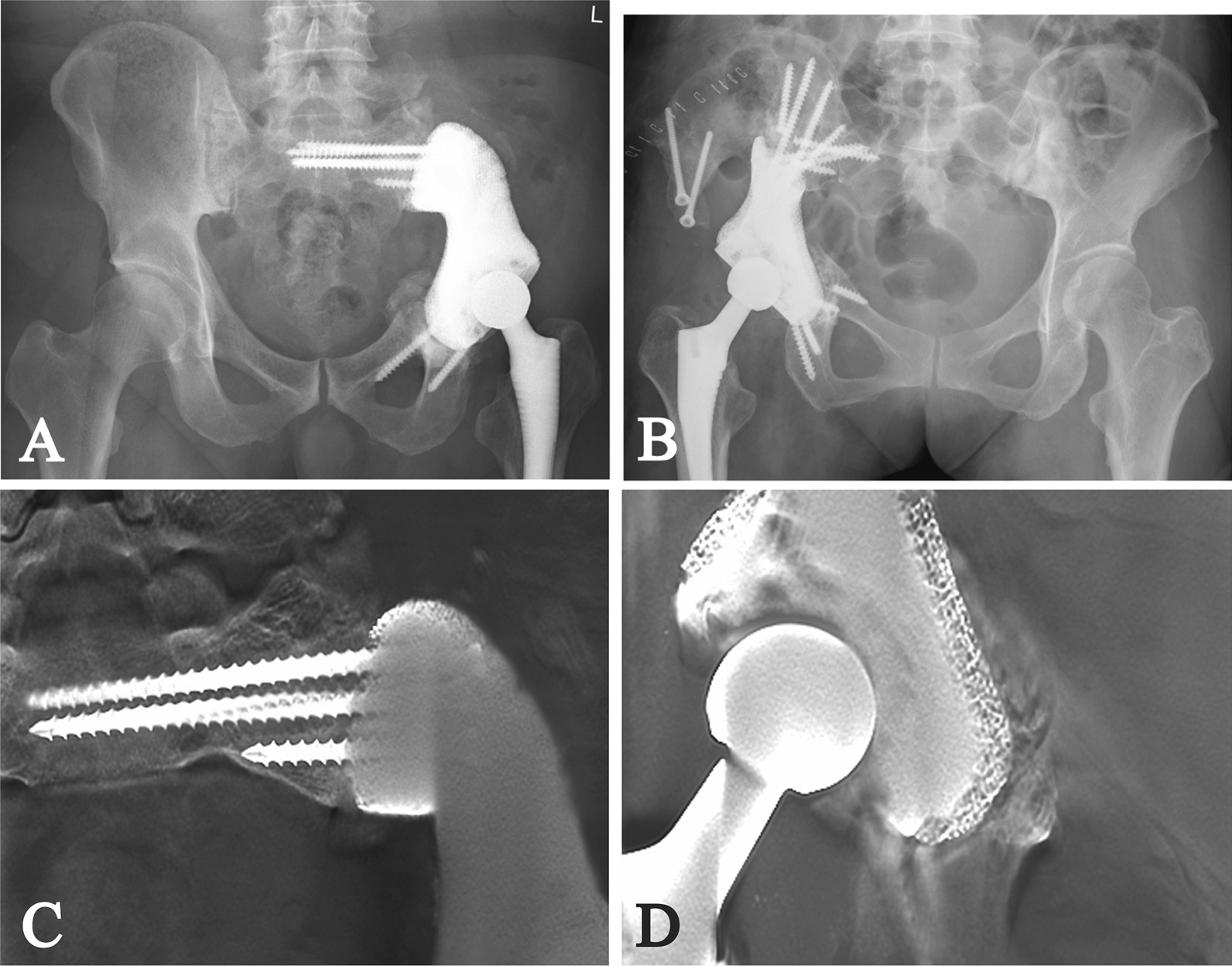
**A**, **B** Two AP plain radiographs of (**A**) patient 1 and (**B**) patient 2 after implantation of custom-made hemipelvic endoprosthesis show planned reconstruction is obtained. **C**, **D** The digital tomosynthesis images show ideal osseointegration in (**C**) patient 1 and (**D**) patient 2

Evaluations, including physical examination and PR of the pelvis, were performed before discharge and monthly in the first 3 months and trimonthly thereafter (Fig. 5A, B). 3D-CT was undertaken before discharge and annually after the surgery. Digital tomosynthesis (Sonialvision Safire II, Shimadzu, Kyoto, Japan) of the pelvis was performed every three months (Fig. 5C, D).

### Primary and secondary study endpoints

Our primary endpoint was the efficacy of 3D-printed custom-made hemipelvic endoprosthesis in restoring the natural location of the acetabulum. The bodyweight moment arm (BWMA) and the cup height discrepancy (CHD) were measured after primary surgery, before revision, and after revision, respectively. The BWMA was the perpendicular distance from two acetabular rotation centers to the symmetry axis of the pelvis on the PR of the pelvis. The CHD was the discrepancy of the perpendicular distance from the horizontal line connecting ischial tuberosities to two acetabular rotation centers on the PR of the pelvis. On the affected side, a greater value of acetabular height than the contralateral side was given a negative value, resulting in a decrease in limb length.

Our second endpoint of interest was the lower-limb function. The function was evaluated six months after primary surgery, before revision, and the most recent follow-up. The 1993 version of the Musculoskeletal Tumor Society (MSTS-93) scale and Harris hip score (HHS) were assessed through chart review, which was carried out by a surgeon not involved with patient care [19, 20]. The MSTS-93 provides a limb-specific assessment based on six categories specific to the entire lower limb (pain, function, emotional acceptance, supports, walking ability, and gait). Each category is scored from 0 to 5 with a total score from 0 to 30 (a higher score being desirable). The HHS is scored from 0 to 100 (a higher score indicating better function). The ambulation distance of each patient was recorded.

Our third endpoint was complications. Complications including deep infection, hip joint dislocation, endoprosthesis fracture, nerve palsy, and vascular incidents were recorded through chart review. Whether aseptic loosening occurred or well-osseointegrated was assessed with digital tomosynthesis. Two senior surgeons independently evaluated digital tomosynthesis scans of the pelvis at the most-recent follow-up evaluation. Good osseointegration would be assessed if we observed the connection between trabecular structures and implant surface.

### Statistical analysis

Descriptive data were provided as median value and range.

## Results

### Acetabulum location

The acetabulum locations were deemed reasonable. Before the revision, the BWMA of the affected side decreased from a median of 10 cm (range 9–11) after primary surgery to 8 cm (range 6–9); the CHD increased from a median of 10 mm (range 0–16) after primary surgery to 14 mm (range 11–23). After the revision, the median BWMA and CHD were 10 cm (range 10–11) and 8 mm (range 2–10), respectively (Table 2).

**Table 2:** Detailed radiographical measurement of acetabular location

Patient	BWMA (cm)			CHD (mm)		
	PoP	PrR	PoR	PoP	PrR	PoR
1	10	9	7	9	13	9
2	10	8	11	12	15	10
3	9	7	11	11	23	8
4	9	8	10	16	16	4
5	11	9	11	9	11	8
6	10	6	10	10	14	9
7	10	8	10	10	11	2
Median	10	8	10	10	14	8

*BWMA* Body Weight Moment Arm, *CHD* Cup Height Discrepancy, *PoP* Post-primary surgery, *PrR* Pre-revision, *PoR* Post-revision

### Function

The deterioration of function was observed during the follow-up after primary surgery, and the improvement of function was observed after revision. Before the revision, the MSTS-93 score decreased from a median of 18 (range 16–19) after primary surgery to 11 (range 8–13). The HHS score dropped from a median of 70 (range 55–77) after primary surgery to 22 (range 21–24). After the revision, the MSTS-93 score improved from a median of 11 (range 8–13) before the revision to 21 (range 18–23). The HHS score improved from a median of 22 (range 21–24) before the revision to 78 (range 75–82) (Table 3). All patients were able to ambulate unsupported for at least 1000 m without pain.

**Table 3:** Detailed function data

Patient	MSTS-93 score			HHS		
	PoP	PrR	PoR	PoP	PrR	PoR
1	18	8	19	72	22	78
2	16	9	21	55	22	79
3	17	8	18	70	21	75
4	19	13	23	77	23	82
5	16	13	22	61	24	81
6	18	12	21	74	22	78
7	19	11	19	58	22	75
Median	18	11	21	70	22	78

*MSTS* Musculoskeletal Tumor Society, *HHS* Harris Hip score, *PoP* Post-primary surgery, *PrR* Pre-revision, *PoR* Post-revision

### Complications

No surgical complications, infection, dislocation, nerve palsy, or vascular incident were observed in this series. All endoprostheses were well-osseointegrated with no aseptic loosening or endoprosthesis fracture.

## Discussion

Modular hemipelvic endoprosthesis is a preferable choice for some surgeons to reconstruct tumorous bone defects involving the acetabulum and restore acceptable lower-limb function [2, 4, 7, 9–12, 15, 21–29]. Aseptic loosening and fracture of modular hemipelvic endoprosthesis are not rare and require revision, whereas revisions are seldom reported with a rate of 0–16% [10, 15, 25, 26, 30]. The aseptic loosening and fracture of modular hemipelvic endoprosthesis are closely associated with the interrupted evolution between primary stability provided by screw fixation and secondary stability offered by osseointegration. Rigid screw fixation can be achieved by experienced surgeons or with patient-specific instruments in the application of modular hemipelvic endoprosthesis [2, 10, 31]. However, as an inevitable deficit corresponding to the high adjustability of modular hemipelvic endoprosthesis, the fit of the anchor part is usually compromised to obtain satisfactory acetabular location and orientation. Even if the structural femoral head can diminish the mismatch between host bone and anchor part, the consequent defective location and orientation of endoprosthesis and screws do not comply with bodyweight transmission. Accordingly, the shear force at the bone-implant interface finally destroys the weak bone-implant connection and results in aseptic loosening and fracture. Therefore, a 3D-printed custom-made hemipelvic endoprosthesis with a porous structure, anatomical conforming, and integrative design might be viable for these challenging revisions. We found revision with 3D-printed custom-made hemipelvic endoprosthesis can obtain ideal restoration of acetabulum location and acceptable function with no incidence of complication.

The revision following the failure of modular hemipelvic endoprosthesis is complex, and the proper solution is not easy to figure out in each patient. Commonly, comparing to bone defects after resection, the bone defects destroyed by failed endoprosthesis are strikingly irregular. Besides, structural femoral head autografting is unworkable in revision due to the lack of autograft resources [2]. Thus, 3D-printed custom-made hemipelvic endoprosthesis seems more reasonable in this series because of its high compactness to various bone defects; even modified modular hemipelvic endoprosthesis has been reported [23]. During this workflow, interface fit, screw alignment, and endoprosthesis retrieval are essential for a successful revision. Firstly, the streamline rather than highly-matching surface design is applied in our series because the uneven sclerotic bone atypically generates after primary surgery imperils tight contact and bone ingrowth for a highly-matching surface. In this situation, the streamlined body seems more viable thanks to the acetabular reamer. The reamer is commonly used to remove such sclerotic bone and generate a more compatible surface for the endoprosthesis implant. After that, ensuring the endoprosthesis location should be carefully done repeatedly by checking the fit of bone-implant margin, continuity of arcuate line, and artificial acetabulum orientation. Secondly, screw alignment should avoid previous screw tunnels because failing to retrieve the embedded screw can directly result in fixation failure. Even the retrieval procedure goes well; the empty screw tunnels can jeopardize rigid fixation. What’s more, the screw alignment following bodyweight transmission enables better mechanical property [12]. Finally, the retrieval of intact screws is usually accessible with a sudden torsion; however, the challenging part is to retrieve the fractured screws embedding in the host bone. Various instruments, including rongeur, bone file, and forceps, are introduced to excavate a 5 mm deep hole to expose the screw stump. Then the screw stump was clamped and torqued with a sudden vigorous force. This method usually works well in our application; nevertheless, it can fail in patients with good bone conditions. In that case, cutting the tail would be selected rather than retrieving all screws by employing more destructive methods, and the bypass fixation can be optimized during preoperative design because the fixation idea varies the screw distribution between two endoprostheses. In detail, most of the screw passages of the previous modular hemipelvic endoprosthesis distribute in the sacral ala [12], while the screw passages of the 3D-printed custom-made endoprosthesis are distributed in the sacral vestibule, offering the bypass opportunity [18, 32].

The deterioration of function was observed during the follow-up after primary surgery, and the improvement of function was obtained after the revision. Previous hemipelvic reconstruction studies following periacetabular tumor resection have described a functional outcome with an MSTS score ranging from 13 to 25 [2, 9–12, 15, 16, 33]. After primary surgery, a comparable lower-limb function was preserved by modular hemipelvic endoprosthesis in our patients. However, the function scores deteriorated thereafter till the revision. Although patients’ focus might shift from being sarcoma survivors to being functionally impaired individuals, progressive endoprosthesis instability should be a predominant factor [34]. The following revision offered a stable pelvis, and patients regained proper function with a median MSTS score of 21, even higher than the MSTS score after primary surgery. The reasons are believed as follows: (1), stable pelvic ring, natural bodyweight transmission, and rigid bone-implant connection benefit functional restoring; (2), the reduced surgical approach minimizes the surgical disruption to preserved muscles and therefore provided the opportunity for maximal restoration of function; meanwhile, the posterior approach, which is commonly applied in total hip arthroplasty, also helps to reduce surgical disruption; (3), the different rehabilitation program allows early training in selected patients, resulting in better function.

We observed no surgical complications, infection, dislocation, nerve palsy, or vascular incidents in this series. To our best knowledge, the hemipelvic revision is not sufficiently represented in the literature. Hence, specific procedures, comparing to the previous modular hemipelvic endoprosthesis, are undertaken to prevent major complications. In order to diminish the incidence of infection, similar interfacial design and povidone-iodine are utilized [18]; besides, a new posterior approach or reduced approach are applied to minimize separating enormous scar tissue; moreover, the separated scar tissue and bursa covering the previous implant were all removed to ensure well soft tissue coverage. The prevention of dislocation depends on good acetabulum location, constrained acetabular liner, and appropriate muscle tension by adjusting the length of the femoral neck [18]. For the purpose of reducing aseptic loosening, the biocompatibility of the endoprosthesis was enhanced by elevating the matching degree at the contacting area and modifying the configuration of the porous structure [35–38]. Consequently, the pain relief during follow-up, the good walking ability, the stable endoprosthesis without migration, and the radiographic images demonstrated ideal osseointegration. As to preventing endoprosthesis fracture, (1) the reconstruction of the intact pelvic ring improved mechanic distribution around the endoprosthesis; (2) the endoprosthesis strength was reinforced by continuous solid structure inside the endoprosthesis. Finally, a porous structure can disrupt the sciatic nerve; therefore, we polished the sciatic foramen of the endoprosthesis.

This study also had limitations. Firstly, our follow-up is short; unknown drawbacks might occur in long-term follow-up. Secondly, the retrospective, non-comparative design and the small number of patients limited the power of this series. Hence, a more extensive multi-institutional study is needed to compare this approach with other solutions, such as flail hip, hip transposition, and updated modular hemipelvic endoprosthesis.

## Conclusions

Revision with 3D-printed custom-made hemipelvic endoprostheses benefited in reconstructing stable pelvic ring and natural bodyweight transmission for patients encountering the aseptic loosening and fracture of modular hemipelvic endoprosthesis. The revision surgery and appropriate rehabilitation program improved patients’ function to a median MSTS score of 22 and pain-free ambulation. The incidence of the complications was low via this individualized workflow.

## Data Availability

The datasets used and/or analysed during the current study are available from the corresponding author on reasonable request.

## Abbreviations

3D: Three dimensional
BWMA: Bodyweight moment arm
CHD: Cup height discrepancy
MSTS: Musculoskeletal tumor society
HHS: Harris hip score
CT: Computerized tomography
PR: Plain radiograph

## References

[1] Enneking WF, Dunham WK (1978). Resection and reconstruction for primary neoplasms involving the innominate bone. J Bone Jt Surg Am.

[2] Guo W, Li D, Tang X, Yang Y, Ji T (2007). Reconstruction with modular hemipelvic prostheses for periacetabular tumor. Clin Orthop Relat Res.

[3] Sun W, Li J, Li Q, Li G, Cai Z (2011). Clinical effectiveness of hemipelvic reconstruction using computer-aided custom-made prostheses after resection of malignant pelvic tumors. J Arthroplasty.

[4] Steel HH (1978). Partial or complete resection of the hemipelvis. An alternative to hindquarter amputation for periacetabular chondrosarcoma of the pelvis. J Bone Jt Surg Am.

[5] Gebert C, Wessling M, Hoffmann C, Roedl R, Winkelmann W, Gosheger G, Hardes J (2011). Hip transposition as a limb salvage procedure following the resection of periacetabular tumors. J Surg Oncol.

[6] Kunisada T, Fujiwara T, Hasei J, Nakata E, Senda M, Ozaki T (2019). Temporary external fixation can stabilize hip transposition arthroplasty after resection of malignant periacetabular bone tumors. Clin Orthop Relat Res.

[7] Eilber FR, Grant TT, Sakai D, Morton DL (1979). Internal hemipelvectomy–excision of the hemipelvis with limb preservation. An alternative to hemipelvectomy. Cancer.

[8] Gradinger R, Rechl H, Hipp E (1991). Pelvic osteosarcoma. Resection, reconstruction, local control, and survival statistics. Clin Orthop Relat Res.

[9] Zhou Y, Duan H, Liu Y, Min L, Kong Q, Tu C (2011). Outcome after pelvic sarcoma resection and reconstruction with a modular hemipelvic prostheses. Int Orthop.

[10] Ji T, Guo W, Yang RL, Tang XD, Wang YF (2013). Modular hemipelvic endoprosthesis reconstruction—experience in 100 patients with mid-term follow-up results. Eur J Surg Oncol.

[11] Wang B, Xie X, Yin J, Zou C, Wang J, Huang G, Wang Y, Shen J (2015). Reconstruction with modular hemipelvic endoprosthesis after pelvic tumor resection: a report of 50 consecutive cases. PLoS ONE.

[12] Ji T, Yang Y, Tang X, Liang H, Yan T, Yang R, Guo W (2020). 3D-printed modular hemipelvic endoprosthetic reconstruction following periacetabular tumor resection: early results of 80 consecutive cases. J Bone Jt Surg Am.

[13] Liu X, Liu Y, Lu W, Liao S, Du Q, Deng Z, Lu W (2019). Combined application of modified three-dimensional printed anatomic templates and customized cutting blocks in pelvic reconstruction after pelvic tumor resection. J Arthroplasty.

[14] Angelini A, Calabro T, Pala E, Trovarelli G, Maraldi M, Ruggieri P (2015). Resection and reconstruction of pelvic bone tumors. Orthopedics.

[15] Guo Z, Li J, Pei GX, Li XD, Wang Z (2010). Pelvic reconstruction with a combined hemipelvic prostheses after resection of primary malignant tumor. Surg Oncol.

[16] Ogura K, Susa M, Morioka H, Matsumine A, Ishii T, Hamada K, Ueda T, Kawai A (2018). Reconstruction using a constrained-type hip tumor prosthesis after resection of malignant periacetabular tumors: a study by the Japanese Musculoskeletal Oncology Group (JMOG). J Surg Oncol.

[17] Wong KC, Kumta SM, Geel NV, Demol J (2015). One-step reconstruction with a 3D-printed, biomechanically evaluated custom implant after complex pelvic tumor resection. Comput Aided Surg.

[18] Wang J, Min L, Lu M, Zhang Y, Wang Y, Luo Y, Zhou Y, Duan H, Tu C (2020). What are the complications of three-dimensionally printed, custom-made, integrative hemipelvic endoprostheses in patients with primary malignancies involving the acetabulum, and what is the function of these patients?. Clin Orthop Relat Res.

[19] Enneking WF, Dunham W, Gebhardt MC, Malawar M, Pritchard DJ (1993). A system for the functional evaluation of reconstructive procedures after surgical treatment of tumors of the musculoskeletal system. Clin Orthop Relat Res.

[20] Harris WH (1969). Traumatic arthritis of the hip after dislocation and acetabular fractures: treatment by mold arthroplasty. An end-result study using a new method of result evaluation. J Bone Jt Surg Am.

[21] Ji T, Guo W, Tang X-D, Yang Y (2010). Reconstruction of type II+III pelvic resection with a modular hemipelvic endoprosthesis: a finite element analysis study. Orthop Surg.

[22] Zang J, Guo W, Yang Y, Xie L (2014). Reconstruction of the hemipelvis with a modular prosthesis after resection of a primary malignant peri-acetabular tumour involving the sacroiliac joint. Bone Jt J.

[23] Liang H, Ji T, Zhang Y, Wang Y, Guo W (2017). Reconstruction with 3D-printed pelvic endoprostheses after resection of a pelvic tumour. Bone Jt J.

[24] Zhang Y, Tang X, Ji T, Yan T, Yang R, Yang Y, Wei R, Liang H, Guo W (2018). Is a modular pedicle-hemipelvic endoprosthesis durable at short term in patients undergoing enneking type I + II tumor resections with or without sacroiliac involvement?. Clin Orthop Relat Res.

[25] Wang B, Sun P, Xie X, Wu W, Tu J, Ouyang J, Shen J (2015). A novel combined hemipelvic endoprosthesis for peri-acetabular tumours involving sacroiliac joint: a finite element study. Int Orthop.

[26] Wang B, Zou C, Hu X, Tu J, Yao H, Yin J, Huang G, Xie X, Shen J (2019). Reconstruction with a novel combined hemipelvic endoprosthesis after resection of periacetabular tumors involving the sacroiliac joint: a report of 25 consecutive cases. BMC Cancer.

[27] Witte D, Bernd L, Bruns J, Gosheger G, Hardes J, Hartwig E, Lehner B, Melcher I, Mutschler W, Schulte M (2009). Limb-salvage reconstruction with MUTARS hemipelvic endoprosthesis: a prospective multicenter study. Eur J Surg Oncol.

[28] Falkinstein Y, Ahlmann ER, Menendez LR (2008). Reconstruction of type II pelvic resection with a new peri-acetabular reconstruction endoprosthesis. J Bone Jt Surg Br.

[29] Schwartz AJ, Kiatisevi P, Eilber FC, Eilber FR, Eckardt JJ (2009). The Friedman-Eilber resection arthroplasty of the pelvis. Clin Orthop Relat Res.

[30] Ozaki T, Hoffmann C, Hillmann A, Gosheger G, Lindner N, Winkelmann W (2002). Implantation of hemipelvic prosthesis after resection of sarcoma. Clin Orthop Relat Res.

[31] Fang X, Yu Z, Xiong Y, Yuan F, Liu H, Wu F, Zhang W, Luo Y, Song L, Tu C (2018). Improved virtual surgical planning with 3D-multimodality image for malignant giant pelvic tumors. Cancer Manag Res.

[32] Carlson DA, Scheid DK, Maar DC, Baele JR, Kaehr DM (2000). Safe placement of S1 and S2 iliosacral screws: the "vestibule" concept. J Orthop Trauma.

[33] Ueda T, Kakunaga S, Takenaka S, Araki N, Yoshikawa H (2013). Constrained total hip megaprosthesis for primary periacetabular tumors. Clin Orthop Relat Res.

[34] Sherman CE, O'Connor MI, Sim FH (2012). Survival, local recurrence, and function after pelvic limb salvage at 23 to 38 years of followup. Clin Orthop Relat Res.

[35] Ghouse S, Reznikov N, Boughton OR, Babu S, Geoffrey Ng KC, Blunn G, Cobb JP, Stevens MM, Jeffers JRT (2019). The design and in vivo testing of a locally stiffness-matched porous scaffold. Appl Mater Today.

[36] Palmquist A, Snis A, Emanuelsson L, Browne M, Thomsen P (2013). Long-term biocompatibility and osseointegration of electron beam melted, free-form-fabricated solid and porous titanium alloy: experimental studies in sheep. J Biomater Appl.

[37] Karageorgiou V, Kaplan D (2005). Porosity of 3D biomaterial scaffolds and osteogenesis. Biomaterials.

[38] Hara D, Nakashima Y, Sato T, Hirata M, Kanazawa M, Kohno Y, Yoshimoto K, Yoshihara Y, Nakamura A, Nakao Y (2016). Bone bonding strength of diamond-structured porous titanium-alloy implants manufactured using the electron beam-melting technique. Mater Sci Eng C.

